# Placido Sub-Pixel Edge Detection Algorithm Based on Enhanced Mexican Hat Wavelet Transform and Improved Zernike Moments

**DOI:** 10.3390/jimaging11080267

**Published:** 2025-08-11

**Authors:** Yujie Wang, Jinyu Liang, Yating Xiao, Xinfeng Liu, Jiale Li, Guangyu Cui, Quan Zhang

**Affiliations:** School of Information and Communication Engineering, North University of China, Taiyuan 030051, China; wangyujie2512@163.com (Y.W.); liangjinyu_2025@163.com (J.L.); xiaoyating5268@163.com (Y.X.); lxf18735060428@163.com (X.L.); 15340682084@163.com (J.L.); 13848982614@163.com (G.C.)

**Keywords:** Mexican hat wavelet transform, Zernike moments, sub-pixel, Placido image, edge detection

## Abstract

In order to meet the high-precision location requirements of the corneal Placido ring edge in corneal topographic reconstruction, this paper proposes a sub-pixel edge detection algorithm based on multi-scale and multi-position enhanced Mexican Hat Wavelet Transform and improved Zernike moment. Firstly, the image undergoes preliminary processing using a multi-scale and multi-position enhanced Mexican Hat Wavelet Transform function. Subsequently, the preliminary edge information extracted is relocated based on the Zernike moments of a 9 × 9 template. Finally, two improved adaptive edge threshold algorithms are employed to determine the actual sub-pixel edge points of the image, thereby realizing sub-pixel edge detection for corneal Placido ring images. Through comparison and analysis of edge extraction results from real human eye images obtained using the algorithm proposed in this paper and those from other existing algorithms, it is observed that the average sub-pixel edge error of other algorithms is 0.286 pixels, whereas the proposed algorithm achieves an average error of only 0.094 pixels. Furthermore, the proposed algorithm demonstrates strong robustness against noise.

## 1. Introduction

The Placido disc-based corneal topography instrument is a widely utilized optical diagnostic and therapeutic device. It aids ophthalmologists in diagnosing conditions such as keratoconus and other ocular diseases by quantitatively analyzing the morphological changes in the corneal surface [1]. However, the precision of the edge detection in corneal Placido ring images directly influences the accuracy of corneal topography parameters [2]. Conventional pixel-level edge extraction algorithms, including the Sobel operator [3], Roberts operator [4], Laplacian operator [5], and Canny operator [6], exhibit limited performance in detecting edges within corneal Placido ring images. The wavelet transform is extensively utilized in various domains, including signal processing [7] and image processing. The Mexican Hat Wavelet Transform is capable of effectively extracting edge information due to its strong scale adaptability and robust noise resistance [8]. With the advancement of medical standards and the ongoing progress of science and technology, the image edge detection at the ordinary pixel level has become insufficient to meet the requirements of medical imaging and machine vision. In contrast, sub-pixel edge detection technology typically yields superior results. Sub-pixel edge detection algorithms can be categorized into three main types: the fitting method [9], the interpolation method [10], and the moment method [11,12,13,14,15,16,17]. Among the various moment-based methods, Zernike moments are extensively utilized owing to their rotation invariance and robustness against noise. Ghosal et al. [13] first proposed the orthogonal Zernike moment edge operator based on a 5 × 5 template. Zhao et al. [14] proposed a 9 × 9 template Zernike moment operator to further improve the edge refinement ability. Bao [15] proposed a Zernike moment sub-pixel edge detection algorithm combined with the Canny operator to improve the accuracy of edge detection. Kong et al. [16] adopted the Canny operator combined with the Zernike moment sub-pixel edge detection algorithm of the improved edge model. This method has a small error in detecting binary images, but there is a double-edged phenomenon in the detection of corneal Placido ring images. Xie et al. [17] proposed a segmented detection method using the Roberts operator for coarse positioning and Zernike moment for precise positioning, which effectively improved the detection efficiency and accuracy of the algorithm.

**Figure 1:**
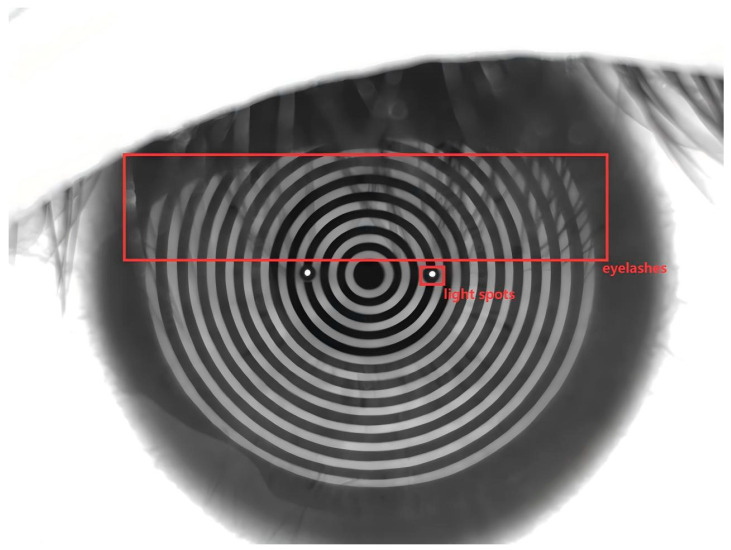
Corneal Placido ring image.

The corneal Placido ring image is presented in Figure 1. It is evident from the box selection section that the interference caused by eyelashes and light spots in the corneal Placido ring image can significantly impair the edge detection of the image, thereby affecting the accuracy of subsequent corneal topography reconstruction.

Therefore, to enhance the accuracy of Placido ring edge extraction, this paper proposes a sub-pixel edge detection algorithm for corneal Placido rings. The algorithm is based on multi-scale and multi-position enhanced Mexican Hat Wavelet Transform, combined with an improved Zernike moment. The enhanced Mexican Hat Wavelet Transform is employed to process the image, thereby eliminating the effects of eyelashes and light spots and obtaining the preliminary edge image. Subsequently, two adaptive threshold segmentation algorithms are applied to filter the sub-pixel edge points of Zernike moments, ultimately yielding the final image edge.

The main contributions of this paper are as follows: (i) a radially modulated Mexican Hat Wavelet, (ii) a multi-scale, multi-position convolution strategy, and (iii) two adaptive thresholding algorithms.

## 2. Enhanced Mexican Hat Wavelet Transform Edge Detection Algorithm

### 2.1. Enhanced Mexican Hat Wavelet Transform

The Mexican Hat Wavelet can precisely identify gray-level variations near the edges of an image due to its second derivative property. Owing to the fixed response characteristic of the traditional Mexican Hat Wavelet, it is challenging to accurately match the target boundaries when addressing targets of varying scales [18]. In light of this, this paper incorporates radial Gaussian modulation into the Mexican Hat Wavelet function to dynamically adjust the functional response. This enhances sensitivity when approaching the target boundary while simultaneously amplifying the response farther from the circular center, thereby achieving adaptability to multi-scale and multi-position responses.

The Mexican Hat Wavelet basis function is defined as (1).

(1) $$h(x,y)=\frac{1}{\sigma^{2}}(2-\frac{x^{2}+y^{2}}{\sigma^{2}})\exp(-\frac{x^{2}+y^{2}}{2\sigma^{2}})$$

The radial Gaussian enhancement term is incorporated into the above equation.

(2) $$g(x,y)=\exp^{-\frac{\left(\sqrt{x^{2}+y^{2}}-R\right)}{2\delta^{2}}}$$

where σ is the scaling factor, δ is the radial selection bandwidth, and *R* is the target radius. The improved enhanced Mexican Hat Wavelet function is constructed as (3).

(3) $$h_{\sigma }(x,y)=\frac{1}{\sigma^{2}}(2-\frac{x^{2}+y^{2}}{\sigma^{2}})\exp(-\frac{x^{2}+y^{2}}{2\sigma^{2}})\cdot \exp^{-\frac{\left(\sqrt{x^{2}+y^{2}}-R\right)}{2\delta^{2}}}$$

**Figure 2:**
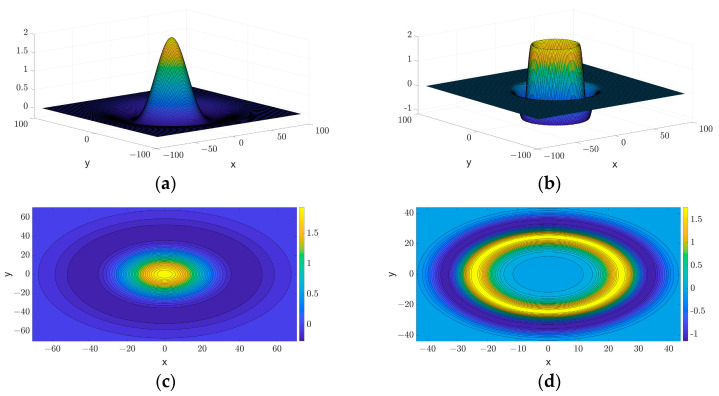
Mexican Hat Wavelet contrast images: (**a**) original MHW 3D image; (**b**) enhanced MHW 3D image; (**c**) original MHW 2D image; (**d**) enhanced MHW 2D image.

Its frequency-domain representation is presented as (4).

(4) $$\begin{array}{cc} H_{\sigma }(u,v)= & 4\pi^{2}\sigma^{2}(u^{2}+v^{2})\exp\left[-2\pi \sigma^{2}(u^{2}+v^{2})\right]\\ & \cdot \exp(-2\pi^{2}\delta^{2}(u^{2}+v^{2}))\cdot J_{0}(2\pi R\sqrt{u^{2}+v^{2}}) \end{array}$$

where $J_{0}(2\pi R\sqrt{u^{2}+v^{2}})$ is the Bessel function modulation. Figure 2 shows the 2D and 3D images of the original and enhanced Mexican Hat Wavelet in the frequency domain. Figure 2 demonstrates that the Mexican Hat Wavelet function, with the incorporation of a radial Gaussian enhancement term, amplifies the response of regions distant from the center of the circle. This enhancement increases the wavelet’s sensitivity to complex edge structures in images, particularly improving its ability to detect weak or blurred edges.

### 2.2. Multi-Scale Multi-Position Image Algorithm

Since the conventional Mexican Hat Wavelet convolution responds only to a single position, it is highly susceptible to noise and local deformation. In order to eliminate the interference caused by eyelashes and light spots in the corneal Placido ring image and extract preliminary edge information from the image, this paper proposes a multi-scale and multi-position Mexican Hat Wavelet convolution algorithm. Specifically, for any given image I(x,y), position steps $b_{x}$ and $b_{y}$ are defined in the horizontal and vertical directions, respectively. At each scale σ, the image cumulative response $S_{\sigma }(x,y)$ at the current scale is obtained after convolution at different positions by translating the wavelet as follows:

(5) $$S_{\sigma }(x,y)=\sum_{b_{x}}\sum_{b_{y}}\left|I*h_{\sigma }(x+b_{x},y+b_{y})\right|$$

The multi-scale cumulative response $S_{\sigma }(x,y)$ of the image is computed by weighting and fusing responses across scales. Thin edges are preserved under smaller extension factor scales, while coarse edges are retained under larger extension factor scales. This process emphasizes contributions from significant scales while suppressing weaker responses. The total response intensity E(x,y) of the image is calculated as follows:

(6) $$\begin{array}{ll} E(x,y) & =\sum_{\sigma }\left[M\cdot S_{\sigma }(x,y)\right]\\ & \;=\sum_{\sigma }\left[\frac{std(\sum_{b_{x},b_{y}}\left|I*h_{\sigma }(x+b_{x},y+b_{y})\right|)}{\sum_{\sigma^{\prime }}std(\sum_{b_{x},b_{y}}\left|I*h_{\sigma }(x+b_{x},y+b_{y})\right|)}\cdot \sum_{b_{x},b_{y}}|I*h_{\sigma }(x+b_{x},y+b_{y})|\right] \end{array}$$

The convolution operation is carried out between the total response intensity E(x,y) of the image and the Gaussian difference enhancement term DoG. By leveraging the difference between Gaussian filters with varying standard deviations, the response intensities of the ring image and the background image are effectively differentiated. This process enhances the contrast between the bright and dark rings in the corneal Placido ring image, thereby effectively highlighting the edge information of the rings. Ultimately, the preliminary edge information $E_{f}(x,y)$ of the image is obtained as (7).

(7) $$\begin{array}{ll} E_{f}(x,y) & =E(x,y)*DoG\\ & =E(x,y)*\left[\frac{1}{2\pi \sigma_{1}^{2}}e^{-\frac{x^{2}+y^{2}}{2\sigma_{1}^{2}}}-\frac{1}{2\pi \sigma_{2}^{2}}e^{-\frac{x^{2}+y^{2}}{2\sigma_{2}^{2}}}\right] \end{array}$$

By comprehensively integrating multi-scale and multi-positional information, the novel algorithm effectively mitigates local deformation interference and noise within images, removes the influence of eyelashes and light spots, distinguishes the ring structure from the background image, and significantly enhances the extraction performance of both strong and weak edges.

## 3. Improved Zernike Moment Sub-Pixel Edge Detection Algorithm

### 3.1. Zernike Moment Edge Detection Principle

The core in Zernike moments is the orthogonal Zernike polynomials. These polynomials are defined within the unit circle. The nth-order Zernike polynomials are shown in (8).

(8) $$V_{nm(\rho ,\theta )}=R_{nm}e^{im\theta }$$

where $e^{im\theta }$ is the angular component, and i is the imaginary unit. n and m belong to integers and satisfy the following conditions: n≥0, $\left|m\right|\leq n$, and $n-\left|m\right|$ is even.

In practice, since the image is discrete, the pixel coordinates need to be normalized to the unit circle; then, the two-dimensional Zernike moments of the image I(x,y) are defined as (9).

(9) $$Z_{n,m}=\frac{n+1}{\pi }\sum_{x}\sum_{y}I(x,y){V_{n,m}}^{*}(r(x,y),\theta (x,y))\cdot W$$

where $\frac{n+1}{\pi }$ is the normalization function, ${V_{n,m}}^{*}(r,\theta )$ is the integral kernel function, and W is the pixel area weight.

**Figure 3:**
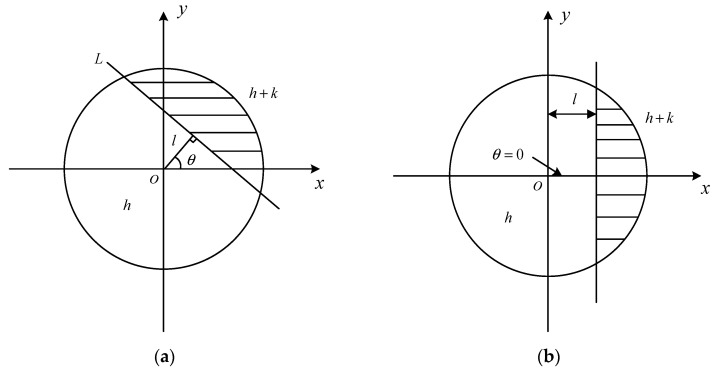
Ideal edge step model of Zernike moment: (**a**) original edge image; (**b**) the rotated edge image.

Zernike moments compute four parameters of pixel points by utilizing orthogonal moments of each order of an image and subsequently determine edge points [19]. Figure 3 illustrates the ideal edge step model of the Zernike moment. In this model, the unit circle represents the region encompassing both the target and background. The portion of line L contained within the unit circle denotes the ideal edge. The gray values on either side of L within the circle are represented by h and h+k, where *k* signifies the gray-level difference. Additionally, l represents the theoretical distance from a point on the circle to the edge, while θ indicates the angle between l and the x-axis.

Because Zernike moments are rotation-invariant, they can be effectively utilized to compute the parameters for edge detection and accurately locate edges even after an image has been rotated. According to Figure 3, the relevant edge parameters can be deduced as (10).

(10) l=Z20Z11′k=3Z11′2(1−l2)32h=1π(Z00−kπ2+ksin−1l+kl1−l2)θ=tan−1(ImZ11ReZ11)

If (x,y) is the determined edge point, considering the template effect, the edge sub-pixel coordinates $\left(x_{s},y_{s}\right)$ corresponding to this point are defined as (11).

(11) $$\left[\begin{array}{l} x_{s}\\ y_{s} \end{array}\right]=\left[\begin{array}{l} x\\ y \end{array}\right]+\frac{Nd}{2}\left[\begin{array}{l} \cos\theta \\ \sin\theta \end{array}\right]$$

where N is the template size.

### 3.2. Double Adaptive Threshold Zernike Moment Sub-Pixel Edge Detection Algorithm

According to the template size effect on Zernike moments, an increase in template size leads to higher accuracy in image edge detection. However, this results in a decrease in detection speed. By balancing accuracy and speed, this paper employs a 9 × 9 template Zernike moment combined with the “coarse-to-fine” positioning detection approach.

In the conventional Zernike moment algorithm, the edge point judgment conditions are defined as $k\geq k_{t}$ and $l\leq l_{t}$, where $k_{t}$ represents the gray-level threshold for judgment, and $l_{t}$ denotes the distance threshold for judgment. $k_{t}$ exhibits a wide range of variations, is highly susceptible to noise, and poses challenges in handling gradual edges. $l_{t}$ represents the constant value $\sqrt{2}/N$ in the edge point judgment condition, and its positioning for complex or irregular shape edges is precise. The conventional approach of manually selecting the threshold requires manual adjustment of the threshold, making it challenging to accurately obtain edge positions across different images. Xie et al. [17] and Huang et al. [20] introduced the Otsu thresholding method for determining the image segmentation threshold $k_{t}$. Specifically, this approach segments an image into foreground and background by leveraging the gray-level values of the image. The method aims to maximize the between-class variance of the gray-level distribution between the target and background, thereby identifying the optimal segmentation threshold. This method relies on the uniformity and prior knowledge of the image, but it is not effective for images with complex backgrounds or high levels of noise. However, corneal Placido ring images often suffer from significant eyelash interference. Consequently, this paper proposes two adaptive threshold algorithms for $k_{t}$ and $l_{t}$, respectively, to enhance the accuracy of image edge detection.

#### 3.2.1. An Adaptive Thresholding Algorithm Based on Local Gradient and Variance

The adaptive threshold algorithm, which incorporates local gradient and variance, can effectively capture the local characteristics of an image while reducing sensitivity to noise by leveraging the intrinsic geometric properties of the image. In regions with low contrast or high noise, the algorithm automatically adjusts the threshold to suppress false edges, thereby enhancing the robustness of edge detection. After calculating the gray value k, distance l, and angle θ based on (10), the gradient amplitude $G_{k}$, local mean $\mu_{k}$, variance $\upsilon_{k}$, and standard deviation $\sigma_{k}$ of the image following Zernike moment convolution are subsequently determined as (12).

(12) $$\left\{\begin{array}{l} G_{k}=\sqrt{{\left(Z_{11}^{R}(x,y)\right)}^{2}+{\left(Z_{11}^{R}(x,y)\right)}^{2}}\\ \mu_{k}=\frac{1}{5\times 5}\sum_{m=x-2}^{x+2}\sum_{n=y-2}^{y+2}Z_{20}(m,n)\\ \upsilon_{k}=\frac{1}{5\times 5-1}\sum_{m=x-2}^{x+2}\sum_{n=y-2}^{y+2}{\left(Z_{20}(m,n)-\mu_{k}\right)}^{2}\\ \sigma_{k}=\sqrt{\upsilon_{k}} \end{array}\right.$$

The local statistics of the convolution image are weighted, and dynamic fusion is applied to the gradient, standard deviation, and log variance. Additionally, the threshold is adaptively adjusted to determine the optimal segmentation threshold $k_{t}$, as shown in (13).

(13) $$k_{t}=\frac{G}{2}+\frac{\sigma }{2}+\frac{\ln(1+\upsilon )}{5}$$

#### 3.2.2. An Adaptive Thresholding Algorithm Based on the Hessian Matrix

The adaptive threshold algorithm based on the principal curvature of the Hessian matrix can decrease the tolerance for curved edge or corner regions, thereby avoiding edge overfitting. This algorithm is capable of accurately distinguishing between genuine edges and structural noise while effectively minimizing the impact of interference on edge detection. The second derivative information of the convolved image is computed using the Hessian matrix to derive the matrix H. Subsequently, eigenvalue decomposition is performed on matrix H to obtain the two principal curvatures, denoted as $\lambda_{1}$ and $\lambda_{2}$. The eigenvalue with the largest absolute value signifies the maximum bending strength $l_{k}$ within the local region. The potential for abnormal edge displacement resulting from geometric distortion or noise can be effectively mitigated by $l_{k}$. When the edge corresponds to a flat region, $l_{k}$ approaches approximately 0, allowing the baseline distance threshold to remain unchanged. In cases where the edge represents a high-curvature region, the threshold $l_{t}$ decreases accordingly to filter out spurious edges within the curved area. The optimal distance threshold $l_{t}$ is obtained as shown in (14).

(14) $$\left\{\begin{array}{l} H=\left[\begin{array}{ll} \frac{\partial^{2}I}{\partial x^{2}} & \frac{\partial^{2}I}{\partial x\partial y}\\ \frac{\partial^{2}I}{\partial x\partial y} & \frac{\partial^{2}I}{\partial y^{2}} \end{array}\right]\\ \lambda_{1},\lambda_{2}=eig(H)\\ l_{k}=\max(|\lambda_{1}|,|\lambda_{2}|)\\ l_{t}=\frac{\sqrt{2}}{N+0.2l_{k}} \end{array}\right.$$

If an edge point satisfies both the intensity condition $k\geq k_{t}$ and the geometric condition $l\leq l_{t}$, it can be classified as an image sub-pixel edge point. Accurate sub-pixel edge detection of a corneal Placido ring image can be achieved through two adaptive threshold segmentation methods.

## 4. Experimental Procedures and Result Analysis

### 4.1. Image Acquisition and Algorithm Enhancement Process

#### 4.1.1. Image Acquisition Process

In this study, Placido disk images of real human eyes were captured using the SW6000D corneal topography system (Tianjin Suowei Electronic Technology Co., Ltd., Tianjin, China), with an image resolution of 1280 × 960 pixels. A total of 10 Placido disk images from healthy subjects were collected. The study was approved by the Biological and Medical Ethics Committee of North University of China, and informed consent was obtained from all subjects.

#### 4.1.2. Algorithm Enhancement Process

This paper adopts the “coarse-to-fine” positioning detection method. Since eyelashes, light spots, and the small ring spacing greatly interfere with the corneal Placido ring image, conventional edge detection operators cannot balance noise immunity and accuracy. Therefore, the enhanced Mexican Hat Wavelet is used to initially locate the image edge. After acquiring the coarse edge of the image, the image is convolved using a 9 × 9 Zernike moment template. Accurate sub-pixel edge detection is then achieved through two adaptive threshold segmentation algorithms. This approach ensures that the algorithm maintains high running speed without compromising the detection accuracy of the improved method. The detailed steps are as follows:

(1) First, the image is convolved with the multi-scale and multi-position Mexican Hat Wavelet function introduced in this paper. The cumulative response is weighted and fused to achieve preliminary localization of the “coarse” edge of the image.
(2) A 9 × 9 Zernike moment template is applied to convolve the “coarse” edge, extracting four parameters for each pixel.
(3) The optimal segmentation thresholds $k_{t}$ and $l_{t}$ are determined using the two adaptive threshold segmentation algorithms proposed herein. The values of k and l for the obtained pixels are jointly evaluated to further ascertain whether the pixel qualifies as an edge point and to calculate its sub-pixel coordinates.
(4) Step 2 is repeated until all edge points have been assessed.

**Figure 4:**
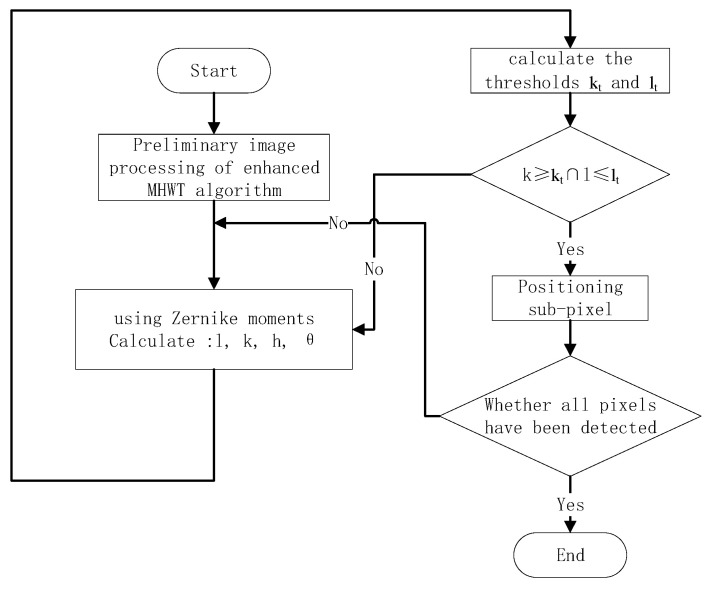
Flowchart of the algorithm.

The flowchart of the algorithm is shown in Figure 4.

### 4.2. Experimental Result Analysis

To validate the superiority and effectiveness of the proposed algorithm, five experimental setups were specifically designed to evaluate its performance in handling eyelash interference, sub-pixel anti-noise capability, multi-scale and multi-position parameter selection, real human eye image processing, and the overall performance enhancement achieved by the improved algorithm. The experimental platform consisted of a Windows 10 operating system, the CPU model was Intel(R) Core(TM) i5-9400, and the proposed sub-pixel edge detection algorithm was implemented using MATLAB R2018b.

#### 4.2.1. The Eyelash Interference Effect

**Figure 5:**
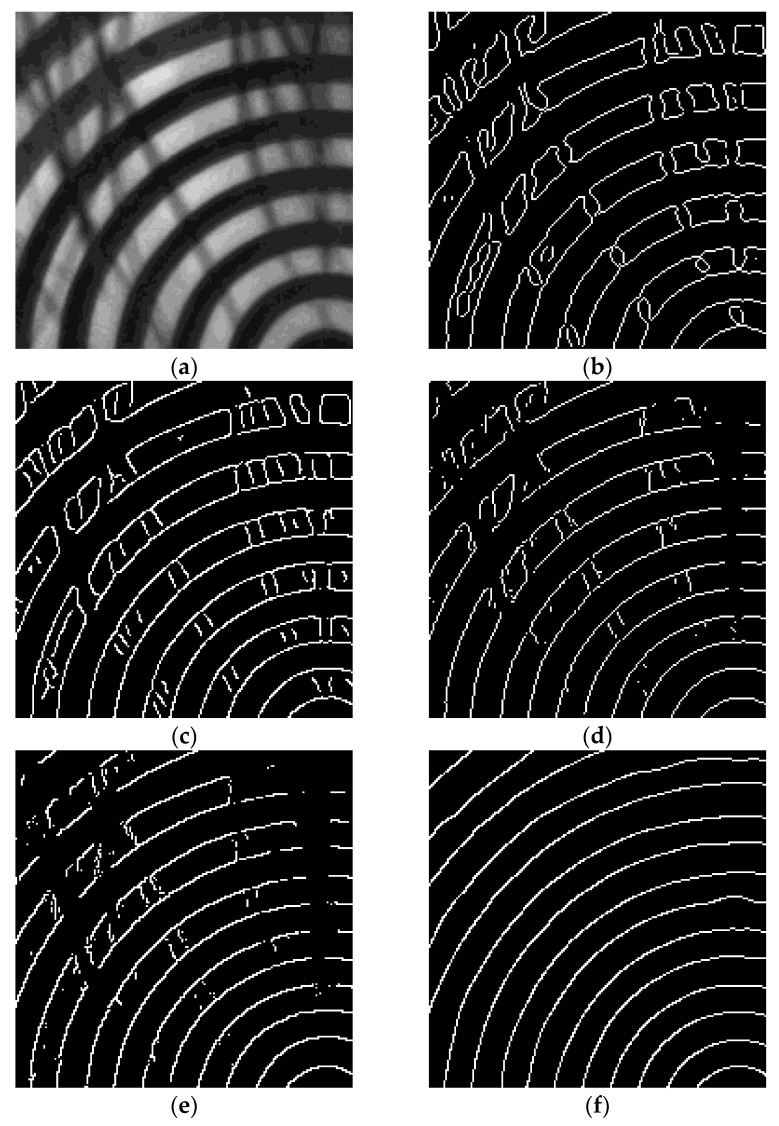
Comparative analysis of preliminary edge detection results across algorithms: (**a**) corneal Placido ring image; (**b**) Laplacian algorithm; (**c**) Canny algorithm; (**d**) Sobel algorithm; (**e**) Roberts algorithm; (**f**) algorithm in this paper.

The aim of the initial set of experiments is to evaluate the performance of the enhanced Mexican Hat Wavelet Transform algorithm proposed in this paper for preliminary edge detection in corneal Placido ring images. The image utilized for this experiment is a 200 × 200 pixels corneal Placido ring image with eyelash occlusion. Figure 5 presents the preliminary edge detection results obtained by each of the comparison algorithms and the proposed algorithm. Experimental comparisons indicate that the Laplacian and Canny operators are significantly affected by eyelash edge interference, while the Sobel and Roberts operators produce fragmented edges in the detection of Placido rings. In contrast, the improved enhanced Mexican Hat Wavelet Transform algorithm demonstrates superior performance compared to the other four methods, exhibiting a strong capability in suppressing eyelash interference.

#### 4.2.2. Accuracy and Noise Immunity of Sub-Pixel Algorithms

**Figure 6:**
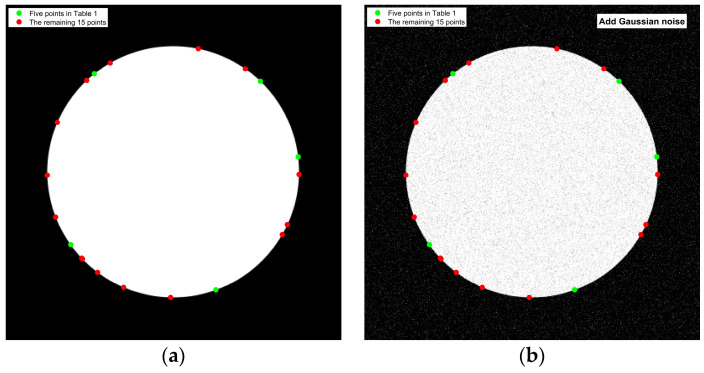
Binary image: (**a**) original binary image; (**b**) binary image with Gaussian noise.

The second set of experiments aimed to evaluate the accuracy and noise robustness of the improved Zernike moment sub-pixel algorithm. A synthetically generated binary image was employed to assess the performance of the enhanced sub-pixel algorithm. The binary image had a resolution of 400 × 400 pixels, with a white circular region centered at coordinates (200, 200) and a radius of 150 pixels, as illustrated in Figure 6a. To evaluate the algorithm’s noise immunity, Gaussian noise with a standard deviation of 0.2 was introduced into the original binary image, resulting in the noisy version shown in Figure 6b.

Twenty points were randomly selected on the circle in Figure 6a. The sub-pixel coordinates of these points were obtained using the improved Zernike-moment-based sub-pixel edge detection algorithm proposed in this paper, and the corresponding coordinate and distance errors were calculated. The coordinate and distance errors derived from the traditional 7 × 7 template Zernike moments, the sub-pixel algorithm proposed in reference [16], the sub-pixel algorithm presented in reference [17], and the interpolation method were compared. A *t*-test was performed on the coordinate errors of all algorithms to calculate the corresponding *p*-value. Due to space constraints, only the coordinates of five selected points are displayed in Table 1 and Table 2.

As shown in Table 1 and Table 2, the average distance errors associated with the traditional 7 × 7 Zernike algorithm and the interpolation method are relatively high. In comparison with the algorithms presented in references [16,17], the algorithms proposed in this paper demonstrate significantly improved accuracy, with an average error within 0.1 pixels. Furthermore, the standard deviation of the proposed algorithm is 0.0687, which is lower than that of the other algorithms. The *t*-test *p*-value is 0.9998, exceeding that of competing algorithms. These results indicate that the sub-pixel edge detection achieved by the proposed algorithm is more stable and exhibits the smallest significant difference from the true coordinates. Consequently, the algorithm enables sub-pixel edge extraction of the Placido disc with an error margin of less than 0.1 pixels.

**Figure 7:**
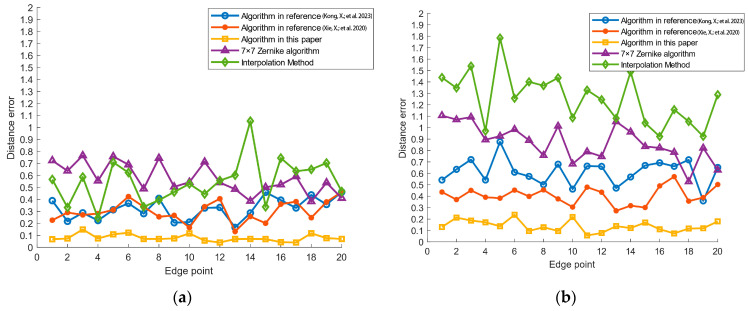
Analysis of distance error variation for each algorithm: (**a**) binary image distance error; (**b**) noisy image distance error [16,17].

To evaluate the noise immunity of the proposed algorithm, 20 points with identical coordinates are selected from Figure 6b. Each sub-pixel coordinate, along with its corresponding coordinate error and distance error, is calculated using the same method. The variation in distance error for each algorithm, comparing the original binary image to the binary image with added Gaussian noise, is presented in Figure 7.

According to the distance error of the noisy image in Figure 7, the distance error of the other three methods becomes significantly larger in the binary image with Gaussian noise, while the proposed algorithm is least affected by noise interference, and the average distance error increases by 0.0736 pixels.

#### 4.2.3. Multi-Scale and Multi-Position Parameter Selection

**Figure 8:**
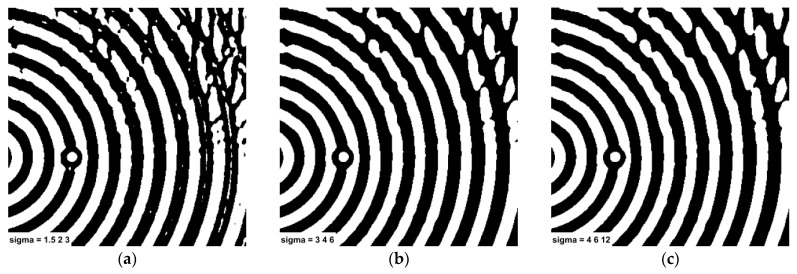
Comparative analysis of various scaling factors: (**a**) σ set to 1.5, 2, and 3; (**b**) σ set to 3, 4, and 6; (**c**) σ set to 4, 6, and 12.

The objective of the third set of experiments is to determine the optimal parameters for multi-scale and multi-position configurations. To determine the optimal scaling factor, three distinct sets of scaling factors were employed to conduct a multi-scale enhanced Mexican Hat Wavelet Transform on Figure 1. The results indicate that smaller scaling factors tend to produce more detailed eyelash-like artifacts in the response image. When the scaling factor exceeds 3, 4, and 6, respectively, the enhancement effect plateaus, with no significant improvement observed. The comparative results are presented in Figure 8.

**Figure 9:**
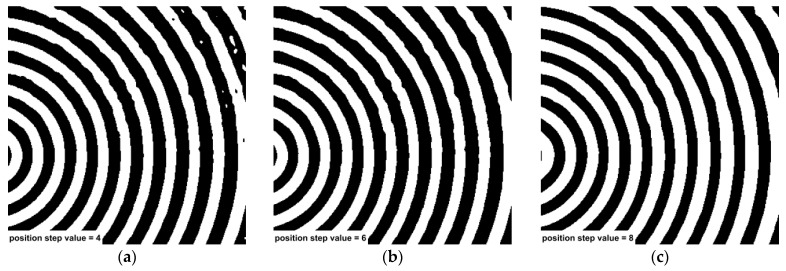
Comparative analysis of various scaling factors: (**a**) position step value set 4; (**b**) position step value set 6; (**c**) position step value set 8.

After determining the appropriate scaling factor, the corresponding optimal position step value was further identified. Three distinct position step values were designed to conduct a multi-scale and multi-position enhanced Mexican Hat Wavelet Transform on Figure 1. The results indicate that both bright spots and eyelashes were effectively suppressed. When the position step value was set to 4, residual spots appeared in the response image, which could interfere with the detection process. When the position step value was increased to 8, the width of the black ring in the response image became narrower, potentially reducing the accuracy of sub-pixel edge detection. The comparative results are presented in Figure 9.

#### 4.2.4. Corneal Placido Image Processing Capability

**Figure 10:**
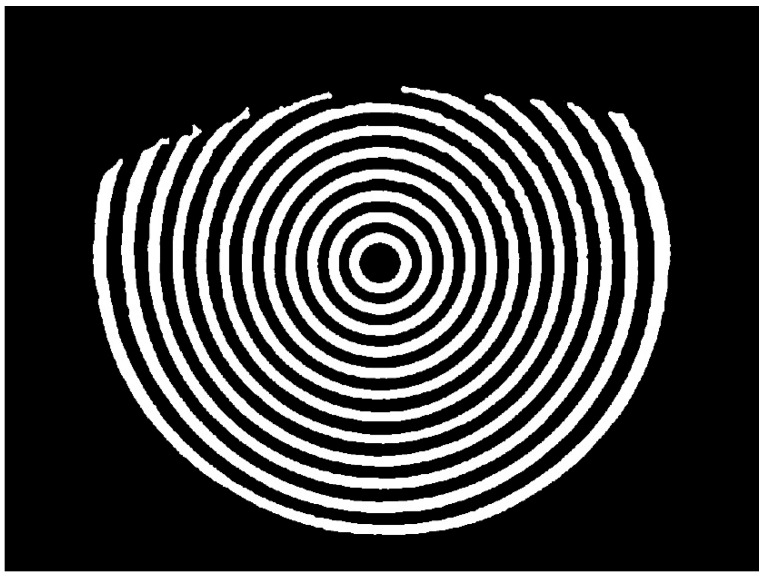
Corneal Placido ring response image.

The objective of the fourth set of experiments is to evaluate the edge extraction performance of the improved Zernike moment sub-pixel algorithm on corneal Placido ring images. With the scaling factor σ set to 3, 4, and 6, and the position step value set to 6, the multi-scale and multi-position enhanced Mexican Hat Wavelet Transform algorithm is applied to process Figure 1. As a result, the response corneal Placido ring image is obtained, as illustrated in Figure 10, with an image resolution of 1280 × 960 pixels.

**Figure 11:**
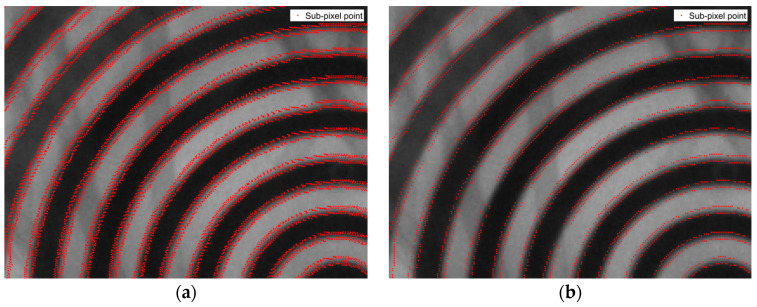

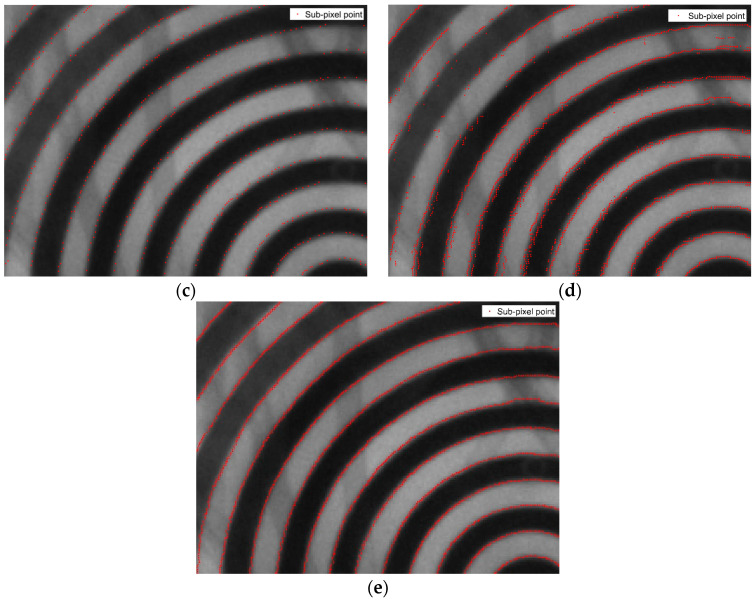
Sub-pixel edge magnification analysis of each algorithm: (**a**) algorithm in reference [16]; (**b**) 7 × 7 Zernike algorithm; (**c**) interpolation method; (**d**) algorithm in reference [17]; (**e**) algorithm in this paper.

The four aforementioned algorithms, along with the improved algorithm proposed in this paper, are applied to perform sub-pixel edge detection on the response image. The resulting sub-pixel edge points are visualized in Figure 1, while the local magnified views illustrating the performance of each algorithm are presented in Figure 11.

**Figure 12:**
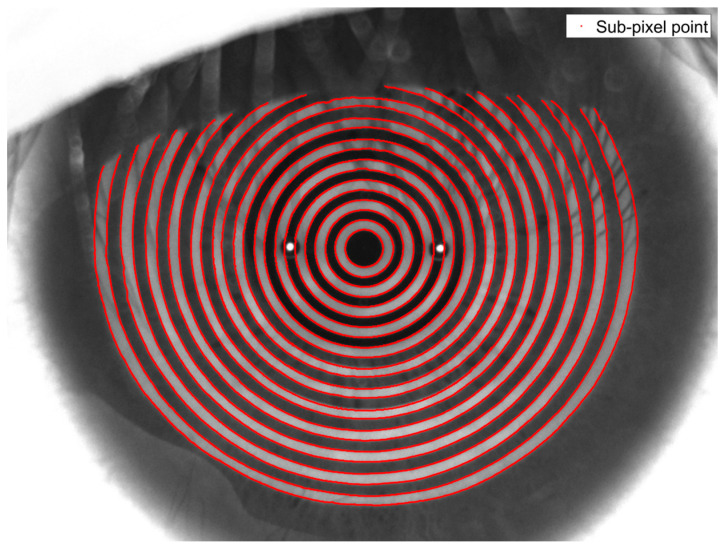
The proposed algorithm Placido ring edge detection results.

Figure 11 demonstrates that the algorithm presented in reference [16] exhibits a significant double-edged phenomenon when detecting Placido ring images, with scattered sub-pixel edge points that do not provide a reliable basis for identifying the actual edge of the Placido ring. Compared to the edge shape obtained using the conventional 7 × 7 template Zernike moment algorithm, the edge shape derived from the sub-pixel edge points is closer to the true edge of the Placido ring; however, the double-edged phenomenon still persists. Although the interpolation method mitigates the double-edged effect, it introduces discontinuities among the sub-pixel edge points, resulting in poor edge connectivity. Although the edge connectivity was enhanced in the algorithm presented in reference [17], the double-edged phenomenon remains present in the resulting edge image. In comparison with the aforementioned four algorithms, the improved algorithm proposed in this paper effectively reduces the double-edged phenomenon, yields more accurate sub-pixel edge point coordinates, and achieves better edge point connectivity. Figure 12 illustrates the enhanced sub-pixel edge detection performance of the proposed algorithm on corneal Placido ring images.

#### 4.2.5. The Performance of the Improved Algorithm

The objective of the fifth set of experiments is to test the performance of the improved algorithm. To evaluate the practical feasibility of the proposed algorithm, the average running time of each algorithm in processing Figure 1 was measured and compared. As presented in Table 3, the running time of the proposed algorithm is significantly shorter than that of the conventional 7 × 7 template Zernike moment algorithm and the method described in reference [16], although it is slightly longer than those of the algorithms in reference [17] and the interpolation method.

To clarify the contribution of each new component in the proposed algorithm, the impact of the multi-scale and multi-position enhanced Mexican Hat Wavelet Transform algorithm and the improved Zernike-moment-based sub-pixel edge detection algorithm on the sub-pixel coordinates in Figure 6a. was sequentially evaluated, using the Mexican Hat Wavelet Transform combined with the 9 × 9 Zernike moment sub-pixel edge detection algorithm as a baseline. The results indicate that the multi-scale and multi-position enhanced Mexican Hat Wavelet Transform algorithm substantially enhances the accuracy of sub-pixel coordinate estimation, while the improved Zernike moment sub-pixel edge detection algorithm effectively suppresses the occurrence of double edges. The individual contributions of each new component are summarized in Table 4.

## 5. Conclusions

This paper proposes a sub-pixel edge detection algorithm based on the multi-scale enhanced Mexican Hat Wavelet Transform and improved Zernike moments, which is applied to edge detection in corneal Placido ring images. The image is convolved using multi-scale and multi-position enhanced Mexican Hat Wavelet Transforms, and the cumulative responses are then weighted and fused to extract preliminary edge information. In this paper, two adaptive thresholding algorithms combined with a 9 × 9 template of Zernike moments are introduced to perform sub-pixel edge detection on the initially detected edges, thereby achieving accurate sub-pixel edge localization. The experimental results demonstrate that, compared to the algorithms presented in references [16,17], the proposed algorithm achieves a 67% reduction in average distance error and exhibits enhanced robustness to noise. The edge detection in the corneal Placido disk image analysis is accurate and reliable. Although the algorithm proposed in this paper demonstrates high accuracy, the limited size of the clinical dataset, and the parameters related to multi-scale and multi-position analysis still depend on manual selection. Future work will focus on developing more advanced algorithms to provide increasingly accurate feature points for subsequent 3D corneal topography reconstruction.

## Figures and Tables

**Table 1:** Table of sub-pixel coordinates for each algorithm.

Detection Method	Actual Coordinates	Sub-Pixel Coordinates	Coordinate Error
7 × 7 Zernike algorithm	(251, 341)	(251.438, 341.576)	(0.438, 0.576)
	(106, 83)	(106.370, 83.519)	(0.370, 0.519)
	(78, 287)	(78.562, 287.520)	(0.562, 0.520)
	(304, 92)	(304.462, 92.308)	(0.462, 0.308)
	(349, 182)	(349.565, 181.495)	(0.565, 0.505)
Interpolation method	(251,341)	(251.327, 341.462)	(0.327, 0.462)
	(106,83)	(106.271, 83.193)	(0.270, 0.193)
	(78,287)	(77.691, 287.499)	(0.309, 0.499)
	(304,92)	(304.179, 91.832)	(0.179, 0.168)
	(349,182)	(348.393, 181.633)	(0.607, 0.367)
Algorithm in reference [16]	(251, 341)	(251.266, 341.284)	(0.266, 0.284)
	(106, 83)	(106.101, 82.808)	(0.101, 0.192)
	(78, 287)	(77.872, 287.258)	(0.128, 0.258)
	(304, 92)	(304.194, 91.888)	(0.194, 0.112)
	(349, 182)	(349.307, 182.061)	(0.307, 0.061)
Algorithm in reference [17]	(251, 341)	(251.156, 341.164)	(0.156, 0.164)
	(106, 83)	(105.762, 83.166)	(0.238, 0.166)
	(78, 287)	(78.208, 287.173)	(0.208, 0.173)
	(304, 92)	(304.113, 91.733)	(0.113, 0.267)
	(349, 182)	(348.814, 182.251)	(0.186, 0.251)
Algorithm in this paper	(251, 341)	(251.023, 341.063)	(0.023, 0.063)
	(106, 83)	(106.046, 83.057)	(0.046, 0.057)
	(78, 287)	(77.878, 287.086)	(0.122, 0.086)
	(304, 92)	(304.051, 91.947)	(0.051, 0.053)
	(349, 182)	(348.919, 182.071)	(0.082, 0.071)

**Table 2:** Table of distance error for each algorithm.

	7 × 7 Zernike Algorithm	Interpolation Method	Algorithm in Reference [16]	Algorithm in Reference [17]	Algorithm in This Paper
Maximum Distance Error	0.765	0.709	0.389	0.312	0.149
Minimum Distance Error	0.555	0.245	0.217	0.226	0.067
Mean Distance Error	0.688	0.487	0.286	0.278	0.094
Standard Deviation	0.3071	0.3628	0.1793	0.1900	0.0687
*p*-value	0.9940	0.9982	0.9984	0.9991	0.9998

**Table 3:** Execution times of each algorithm.

	7 × 7 Zernike Algorithm	Interpolation Method	Algorithm in Reference [16]	Algorithm in Reference [17]	Algorithm in This Paper
Average Execution Times (ms)	653	319	461	322	384

**Table 4:** The impact of each new component in the proposed algorithm.

Algorithm	Mean Distance Error	Standard Deviation	*p*-Value
MHWT + Zernike	0.632	0.2739	0.9981
Multi-scale and Position Enhanced MHWT + Zernike	0.127	0.2183	0.9988
MHWT + Improved Zernike	0.281	0.0917	0.9992
Proposed Algorithm	0.094	0.0687	0.9998

## Data Availability

The original contributions presented in this study are included in the article. Further inquiries can be directed to the corresponding author.

## References

[1] Campos-García M., Huerta-Carranza O., Moreno-Oliva V.I., Aguirre-Aguirre D., Pantoja-Arredondo L.Á. (2024). Corneal topography using a smartphone-based corneal topographer considering a biconical model for the corneal surface. Opt. Contin..

[2] Gomez-Tejada D., Hernández D.M. (2019). A proposal to eliminate the skew ray error in corneal topography using Placido disks images. Proceedings of the Applied Optical Metrology III.

[3] Vaishnavi L.L., Bhavana M., Manideep D.C.M., Venugopal V. (2024). A Fast and Accurate Object Counting using Sobel Edge Detection System for Real–Time Star Gazing. Proceedings of the 2024 Asia Pacific Conference on Innovation in Technology (APCIT).

[4] Mogos G. (2024). Quantum Image Processing Using Edge Detection Based on Roberts Cross Operators. Proceedings of the International Conference on Smart Computing and Communication.

[5] Ma P., Yuan H., Chen Y., Chen H., Weng G., Liu Y. (2024). A Laplace operator-based active contour model with improved image edge detection performance. Digit. Signal Process..

[6] Zhang Z., Zhu Y. (2024). Adaptive Canny edge detection based on fast median filtering. Proceedings of the Fifth International Conference on Computer Vision and Data Mining (ICCVDM 2024).

[7] Umar M., Ahmad Z., Ullah S., Saleem F., Siddique M.F., Kim J.-M. (2025). Advanced Fault Diagnosis in Milling Machines Using Acoustic Emission and Transfer Learning. IEEE Access.

[8] Zhu S., Zeng C., Xu W., Zhu Q. (2023). Research on multidirectional wavelet transform in optical measurement. Proceedings of the Third International Conference on Optics and Image Processing (ICOIP 2023).

[9] Du Z., Zhang W., Qin J., Lu H., Chen Z., Zheng X. (2015). A Novel Subpixel Curved Edge Localization Method. Proceedings of the Intelligent Computation in Big Data Era: International Conference of Young Computer Scientists. In Proceedings of the Engineers and Educators, ICYCSEE 2015.

[10] Qi Y., Wu G., Li Y., Dong Y. (2024). Research on double cubic interpolation combined with Gaussian fitting sub-pixel edge detection method. Proceedings of the 10th International Symposium on Test Automation & Instrumentation (ISTAI 2024).

[11] Du G., Tong Q., Hou L., Yang D., Liang X. (2025). Sub-pixel edge detection method based on canny-franklin moments. Comput. Integr. Manuf. Syst..

[12] Hagara M., Šatka A., Kubinec P., Stojanović R. (2024). Vibration monitoring using sub-pixel edge localization. Proceedings of the 2024 13th Mediterranean Conference on Embedded Computing (MECO).

[13] Ghosal S., Mehrotra R. (1994). Detection of composite edges. IEEE Trans. Image Process..

[14] Zhao B.Y., Qi Y.J. (2012). Improved algorithm for sub-pixel edge detection based on Zernike moments. Adv. Mater. Res..

[15] Bao E. (2022). Improved Canny-Zernike moment-based subpixel edge detection algorithm. Proceedings of the 6th International Conference on Mechatronics and Intelligent Robotics (ICMIR2022).

[16] Kong X., Yi J., Wang X., Luo K., Hu J. (2023). Full-field mode shape identification based on subpixel edge detection and tracking. Appl. Sci..

[17] Xie X., Ge S., Xie M., Hu F., Jiang N. (2020). An improved industrial sub-pixel edge detection algorithm based on coarse and precise location. J. Ambient Intell. Humaniz. Comput..

[18] Singh A., Raghuthaman N., Rawat A., Singh J. (2020). Representation theorems for the Mexican hat wavelet transform. Math. Methods Appl. Sci..

[19] Xiang F., Wang Z., Yuan X. (2013). Subpixel edge detection: An Improved Zernike orthogonal moments method. Proceedings of the 2013 5th International Conference on Intelligent Human-Machine Systems and Cybernetics.

[20] Huang C., Jin W., Xu Q., Liu Z., Xu Z. (2020). Sub-pixel edge detection algorithm based on canny–zernike moment method. J. Circuits Syst. Comput..

